# Biofunctionalization of 3D printed PEEK using integrated cathodic arc plasma coating: a one-step solution to antimicrobial and bioactive PEEK Implant

**DOI:** 10.1007/s10856-025-06971-7

**Published:** 2025-11-21

**Authors:** Jay Phruekthayanon, Marina Kühn-Kauffeldt, Marvin Kühn, Jörg Gregor Diez, Jutta Tübel, Stephan Heller, Rainer Burgkart, Andreas Obermeier

**Affiliations:** 1 Institute for Electrical Energy Systems, University of Bundeswehr Munich, Neubiberg, Germany; 2 Department 310-Surface Technology and Analytics, Wehrwissenschaftliches Institut für Werk- und Betriebsstoffe (WIWeB), Erding, Germany; 3 Institute of Lightweight Engineering, University of the Bundeswehr Munich, Neubiberg, Germany; 4 Clinic for Orthopedics and Sports Orthopedics, Klinikum rechts der Isar, TUM School of Medicine and Health, Technical University of Munich, Munich, Germany

## Abstract

This study introduces a novel hybrid additive manufacturing (AM) approach that integrates a surface coating process directly into the AM workflow. By incorporating a vacuum arc plasma source into a Fused Filament Fabrication (FFF) system, we combine the design freedom and scalability of 3D printing with the ability to biofunctionalize the printed polymer part in a single fabrication step. Polyetheretherketone (PEEK) is widely used in biomedical engineering due to its excellent mechanical properties, biocompatibility, and radiolucency. However, its bioinert nature poses challenges for infection prevention and bone integration. This study aims to evaluate the coatings produced by this integrated process on a PEEK substrate specifically in a biomedical context, focusing on their antimicrobial performance and cytocompatibility. The results show that zinc (Zn) is the most effective antimicrobial agent among the tested coatings (Ag₂O, Cu, and Zn), achieving a reduction in bacterial adhesion of over 4 log. Moreover, TiO₂/Zn composite coatings exhibit strong antimicrobial activity while maintaining good cytocompatibility with fibroblastic cells in vitro. Qualitative imaging also indicates improved osteoblast attachment on surfaces coated with TiO₂ and TiO₂/Zn. This hybrid manufacturing platform enables the production of implants with tailored structural and biological properties in a single step, representing a significant advancement in the development of next-generation medical implants.

**Graphical Abstract:**

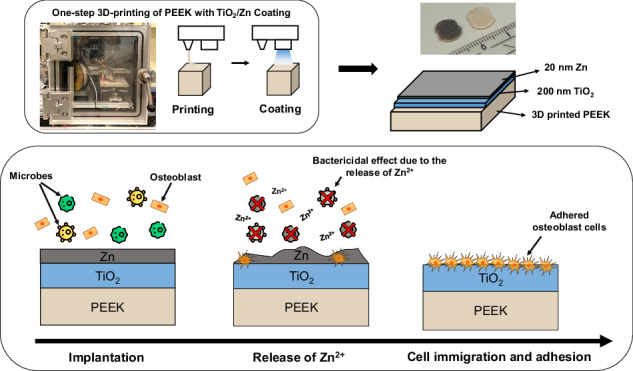

## Introduction

In recent decades, the advent of additive manufacturing (AM) has revolutionized the field of biomedical engineering. Polyetheretherketone (PEEK) has emerged as a prominent material especially in orthodontic and orthopedic implants [1, 2]. Today, using 3D printing technologies, PEEK can be fabricated into customized and individualized implants at a low cost and with high reproducibility [3]. However, enhancing the functionality of PEEK implants to prevent infections and promote integration with bone tissue remains a challenge.

Spinal reconstruction provides a compelling example of where enhanced PEEK implants could be particularly beneficial. Infections and degradations of the spine such as spondylodiscitis serve as a prime example of a condition that could benefit from these advanced implants. In Germany, spondylodiscitis affects 11 out of 100,000 individuals in 2021, a figure that has doubled since 2005, indicating a rising trend of the disease [4]. Studies report a mortality rate for spondylodiscitis of around 5–7% within the first 30 days and 19–22% in the first year [5–7]. The most common bacterial pathogen that cause the infection in the spine is of the staphylococcal species, such as Staphylococcus aureus (*S. aureus*) [5, 8]. The surgical treatment involves reconstruction of the spinal structure by using bone grafts along with synthetic materials such as titanium alloy or PEEK [9, 10]. Such spinal instrumentation procedures are associated with 1–12% infection rate [8]. Additionally, the incidence rate of spinal fusion in Germany is predicted to increase by 83–120% by 2060 [11].

Consequently, it is urgently necessary for implantable materials to possess antimicrobial properties to mitigate the risk of severe postoperative infections. Over the decades, various attempts have been made to create antimicrobial implants. These solutions often involve implant surface modification, such as coatings with antibiotics, nano-silver, antimicrobial enzymes, or peptides [12, 13]. One interesting method to generate a coating on PEEK is the pulsed vacuum arc due to its ability to deposit well adherent thin metallic coatings [14, 15]. This method generates a metallic plasma at the face of the cathode within a high vacuum environment. The highly ionized plasma consisting of evaporated cathode material is accelerated away from the cathode surface and subsequently deposited onto the substrate. The operation of vacuum arcs in pulsed mode [16] enables coating of temperature sensitive substrates such as polymers. By integrating vacuum arc source into a 3D printing system, a new manufacturing method for medical implants emerges, where the printed structure can be subsequently or simultaneously coated in a one-step fabrication as demonstrated in a previous work [17]. Additionally, the use of a vacuum could potentially provide the advantage of improved mechanical properties of the part [18].

Various metals and metal alloys are commonly used in medicine. Titanium (Ti) is widely used as a material for load-bearing implants not only due to its high strength-to-weight ratio but also its passivation layer of titanium oxide (TiO_2_), which reduces the risk of corrosion and increases its ability to bond with native bone [19]. The antimicrobial effect of many metals such as silver (Ag), copper (Cu) and zinc (Zn) has been known historically for decades and is currently experiencing a renaissance in medicine due to the rise of antibiotic-resistant bacteria strains [20, 21]. Zn—a natural trace element and an essential nutrient for the human body—plays a vital role in numerous physiological functions including bone growth [22–27]. Moreover, the antimicrobial effect of Zn, especially in form of nanoparticles (NP), has been proven in various studies [25, 27, 28]. Therefore, using Zn as an antimicrobial agent in an implant material could be of great benefit.

We have developed a prototype of a novel hybrid 3D printer that allows for printing and coating in one single step, eliminating the need for additional handling or post-processing. This study aims to investigate the manufacturing of thin film metal and metal oxide coatings on 3D-printed PEEK surfaces using the hybrid manufacturing method. The coatings were evaluated for their antimicrobial efficacy with a quantitative bacterial adhesion assay as well as qualitative image observation. Furthermore, biocompatibility and adhesion of osteoblasts on such surfaces are demonstrated.

**Fig. 1:**
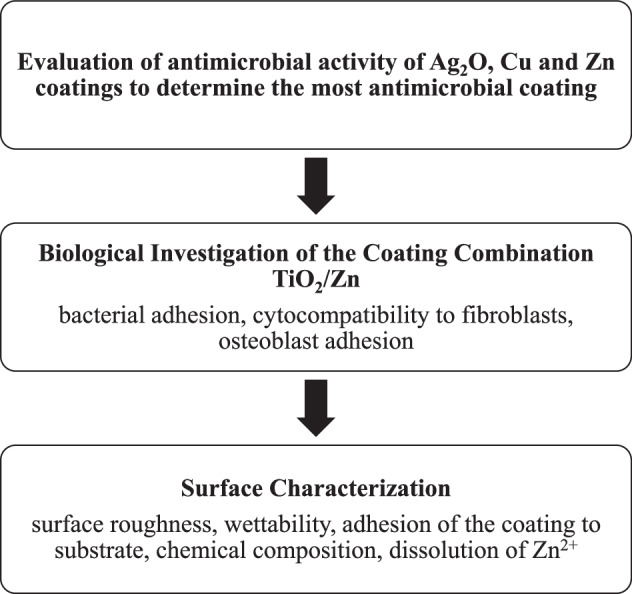
Workflow illustrating the three main steps of the experimental approach: screening of the antimicrobial activity of different coatings, biological investigation of the TiO₂/Zn coating combination, and surface characterization

In this study, we aim to provide insights into the potential advantages and applications of these surfaces in reconstructive surgeries, particularly for preventing implant-associated infections. The corresponding workflow is illustrated in Fig. 1. The results presented herein demonstrate the transformative potential of this approach in developing next-generation medical implants designed to address the evolving needs of modern healthcare.

## Materials and methods

### Specimen preparation

**Fig. 2:**
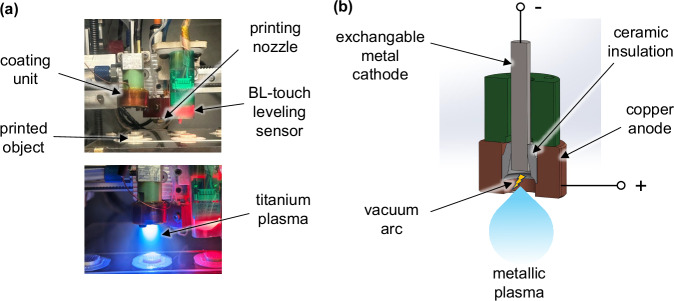
**a** Print head of the custom FFF 3D-printer with integrated coating unit (top). A 3D-printed PEEK specimen being coated with a titanium plasma directly after printing (bottom). **b** CAD assembly shows components and general principle of the coating unit

A custom-built prototype FFF 3D printer, capable of operating under high vacuum conditions, was used to fabricate PEEK substrates. In this work, the print head, as shown in Fig. 2, integrates a coating source, allowing for in situ application of coatings during or immediately after the printing process. All elements of the printer are designed to function in high vacuum environment and the critical elements are additionally cooled with a water-cooling system. The printer design is described in detail in a previous work [29].

Before the print, PEEK filament (W2 Filaments, Frohnleiten, Austria) was bake-dried at 200 °C for 1 h to minimize moisture content, which is critical for achieving high-quality prints. This practice is also beneficial for the sterility of the end product as well as the fabrication chamber. After baking, the filament is stored under high vacuum (10^−3 ^mbar). All printing and coating parameters are summarized in Table 1. Different specimen types were fabricated for different measurements and assays. The CAD model and dimensions of each specimen type are summarized in Fig. S1.

**Table 1:** Printing and coating parameters for the specimen preparation

Process Parameters			
3D-printer		Coating unit	
Extruder temperature	370 °C	Arc current	180 A
Bed plate temperature	160 °C	Pulse duration	400 µs
Printing speed	5–15 mm/s	Pulse frequency	1 Hz
Nozzle diameter	0.4 mm	Cathode materials	Ag, Cu, Ti, Zn
Layer height	0.25 mm	Coatings	Ag_2_O, Cu, TiO_2_, Zn
Extrusion width	0.45 mm		
Infill pattern	Aligned rectilinear		
Infill density	100%		

The thin film coatings were applied immediately after the printing process using the integrated vacuum arc plasma source. The printing and coating process of metals take place at vacuum pressure of approximately 10^−3 ^mbar or lower. For formation of oxide coatings, an oxygen gas (Linde AG, Pullach, Germany) was introduced into the chamber and the pressure was maintained during the coating procedure at approximately 2.5 × 10^−1 ^mbar, which ensured sufficient flow of oxygen for formation of oxide coatings with desired stoichiometry [14]. The thickness of the deposited coatings is controlled by the number of plasma pulses, with each material possessing its own unique deposition rate. The deposition rates are determined prior to this study in separate preliminary studies. An exemplary measurement of coating thickness and the resulted average deposition rates of the coatings are shown in Fig. S2 and Table S1, respectively.

Prior to all biological tests, all specimens underwent dry heat sterilization at 120 °C for 3 h to ensure sterility. Dry heat was selected to avoid compromising the characteristics and chemical composition of the coatings.

### Biological investigations

#### Bacterial adhesion

To assess the antimicrobial efficacy of the coatings, a bacterial adhesion assay was conducted. First, a range of potential antimicrobial coatings was tested to identify the most effective material and determine the minimum coating thickness required for antimicrobial efficacy against the target pathogen. The first array of coatings included Ag_2_O, Cu and Zn each at 10, 20, and 50 nm thickness. In this experiment, the antimicrobial efficacy of different metals at different thicknesses was determined. In the second experiment, the strongest antimicrobial material (Zn) was combined with TiO_2_ coating and tested again for its antimicrobial efficacy.

**Fig. 3:**
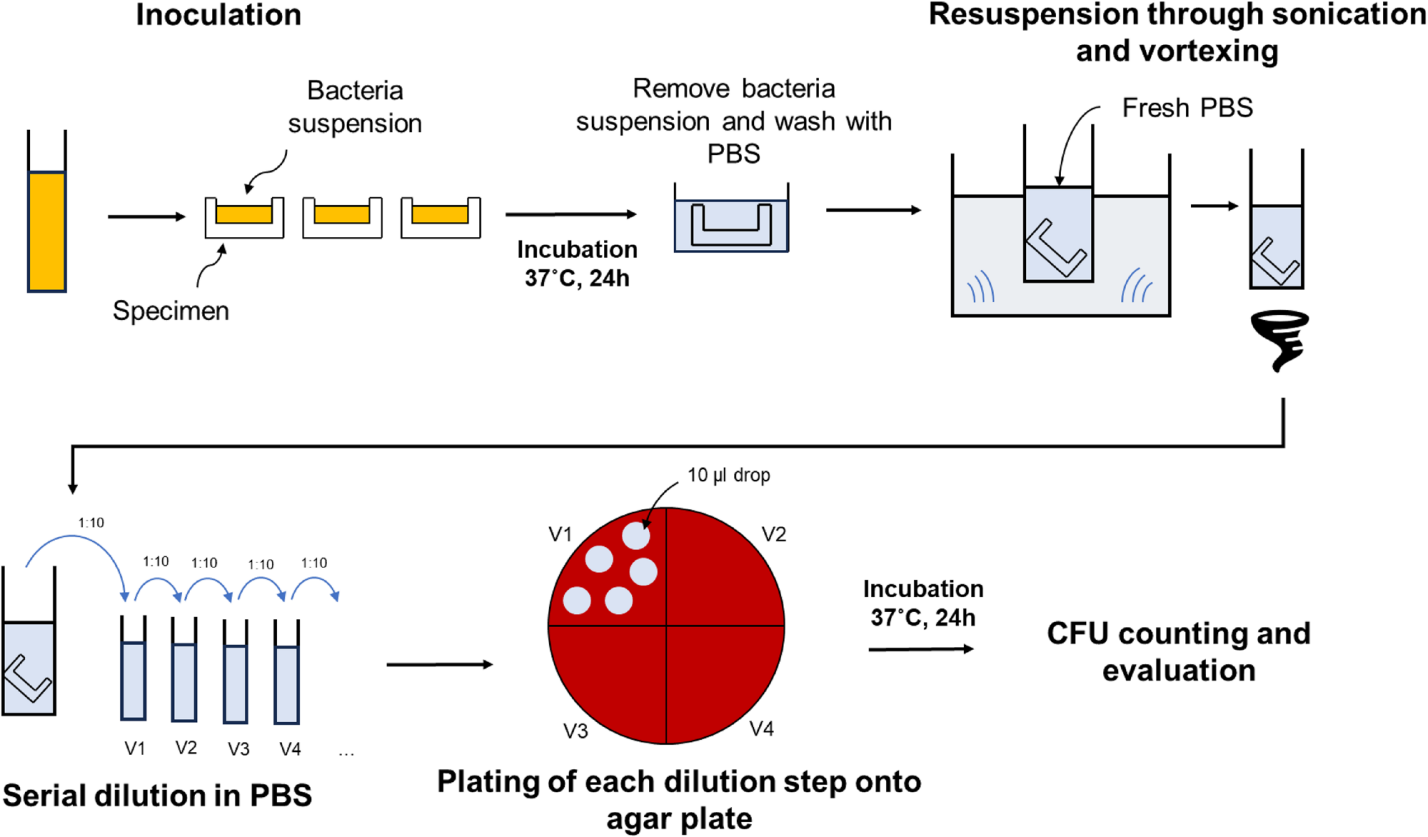
Schematics of the procedure for antibacterial adhesion assay used in this study

The assay for bacterial adhesion followed an adapted ISO 22196, which is established and described in previous works [25, 30] and shown in Fig. 3. The test used *S. aureus* (ATCC 25923) as the bacterial pathogen and bare (uncoated) 3D-printed PEEK surface as reference. The bacteria were pre-cultured on blood agar plates (BD BBL™ Columbia Agar with 5% sheep blood). Utilizing the advantages of 3D printing, the specimens were designed with a bowl-shaped geometry capable of holding approximately 200 µL of bacterial inoculum as illustrated in Fig. 3. The bacterial inoculum was prepared in tryptic soy broth (TSB) and adjusted to an optical density of OD_600_ = 0.01 ( ± 0.005), then incubated on the specimens overnight to allow for bacterial adhesion onto the surface. After incubation, the samples were gently washed with phosphate buffer solution (Merck KGaA, Darmstadt, Germany) with concentration of 1x by volume (1x PBS) to remove any loosely adhered bacteria and TSB residues from the surface. Each specimen was then placed in fresh PBS and subjected to sonication (3 min at 35 kHz) and subsequent vortexing (5 s) to dislodge the adhered bacteria, creating a bacterial suspension. The resulting suspension was subsequently diluted and plated on blood agar plates for colony counting. A spot plating method is used, where 5 × 10 µL drops of each dilution step are placed on to the agar plate and one agar plate can contain up to 4 dilution steps divided into quadrants, as shown in Fig. 3. After 24 h incubation, colony-forming units (CFU) on the agar were counted to obtain quantitative results. The antimicrobial efficacy was then calculated by comparing the CFU on coated versus uncoated PEEK surfaces, expressed as log reduction:

$${Log\; reduction}={\log }_{10}\left(\frac{{{CFU}}_{{uncoated\; PEEK}}}{{{CFU}}_{{coating}}}\right)$$

Additionally, the adhere bacteria on the surfaces are qualitatively evaluated using VK-X3000 confocal laser scanning microscope (LSM) (Keyence, Neu-Isenburg, Germany). For this purpose, small flat specimens of 10 mm diameter were inoculated with a small drop (100 µL) of *S. aureus* suspended in TSB (OD_600_ = 0.01) for 6 h. The specimens were then gently washed once with PBS to remove planktonic bacteria. The adhered bacteria were fixed by incubating the discs in 4% paraformaldehyde for 30 min. Subsequently, the discs were washed twice with PBS and once with distilled water to prevent the formation of salt crystals from PBS residues. Images were acquired using LSM at three randomly selected areas on the surface of each sample.

#### Cytotoxicity to fibroblastic cells

##### Sample and cell culture preparation

The cytotoxicity of the coatings is evaluated in accordance with standard ISO 10993-5 (Biological evaluation of medical devices—Tests for in vitro cytotoxicity). Here, specimens in form of small discs with 10 mm diameter were tested with indirect WST-1 and LDH assay as well as qualitative analysis with LIVE/DEAD® staining. In the indirect method, cells are not in direct contact with the samples; instead, the samples are incubated in culture medium for 24 h, and the resulting extract is used to culture fibroblasts. The indirect method was chosen instead of direct method (seeding the cells directly on the specimen surface) due to the subsequent imaging with fluorescence microscope for LIVE/DEAD® staining. PEEK substrate has been shown to produce high autofluorescence, rendering the imaging of cells directly seeded on the sample with fluorescence microscope ineffective.

For the extraction process, sterilized samples were incubated in a well plate with 2.5 mL of RPMI-1640 extraction medium (Biochrom GmbH, Berlin, Germany) for 24 h. In parallel, L929 mouse fibroblasts (DSMZ GmbH, Braunschweig, Germany; ACC 2)—a well-established cell line for cytotoxicity testing – were seeded in a separate well plate at a density of 20,000 cells per well in 0.4 mL RPMI-1640 medium using 96-well plates.

After the 24 h incubation period, inoculation was performed by transferring the RPMI-1640 medium from the sample extraction wells to the wells containing the prepared cell cultures. The incubation was carried out for an additional 24 h at 37 °C. After the incubation period, cell viability was investigated using WST-1 assay, LDH-Glo™ assay and Live/Dead Staining.

##### WST-1

WST-1 assay was used to measure cell metabolic activity and thus cell viability. The water-soluble stable tetrazolium salt (WST-1) reagent (Roche Holding GmbH, Basel, Switzerland) was then added to the culture medium at a ratio of 1:10. The WST-1 is reduced to soluble formazan by complex cellular mechanisms in living cells. The amount of formazan directly correlates with the number of metabolically active cells and was measured with Multiskan Ascent UV-Vis-Spectrometer (Thermo Fisher Scientific, Massachusetts, USA) at 450 nm with a reference wavelength of 620 nm. Positive control is fibroblast cells in pure (unconditioned) medium.

##### LDH-Glo™

The LDH-Glo™ assay measures the amount of LDH (Lactate dehydrogenase) released into the culture medium from damaged cells. Here, similar to WST-1, the culture medium was mixed with the LDH-Glo™ detection reagent, which generates a luminescent signal proportional to the amount of LDH present. The detection reagent was prepared by mixing 5 mL of LDH detection enzyme with 25 µL of reductase substrate (Promega Corporation, Madison, USA). Subsequently, 50 µL of the prepared detection reagent was added to each well. The enzyme reaction took place under light exclusion during an incubation of 60 min at room temperature. Luminescence was measured using a plate reader Fluoroskan Ascent FL (Thermo Fisher Scientific, Massachusetts, USA). Positive control is fibroblast cells in medium with addition of 0.1% Triton X-100 (Sigma-Aldrich, St. Louis, USA) for complete lysis.

##### Live/dead staining

Lastly, the fibroblast cells incubated with the conditioned media were stained with LIVE/DEAD® Viability/Cytotoxicity Kit (Thermo Fisher Scientific, Waltham, USA). The staining solution is prepared under light exclusion from 10 mL PBS, 5 μL Calcein-AM, and 20 µL Ethidium Homodimer. After preparing the staining solution, the extract was removed from the 96-well plate, and the wells were washed twice with PBS. Subsequently, 250 µL of the staining solution was added to each cell sample. The samples were incubated for 15–20 min in the dark. Finally, the cells were examined under the Axiolab 5 fluorescence microscope (Carl Zeiss, Oberkochen, Germany).

#### Osteoblast adhesion

Prior to this work, primary human osteoblasts (phOB) were isolated from bone tissue during joint replacement surgeries and frozen for future use. The osteoblasts were isolated using the explant method [31] from the spongiosa of femoral heads during a joint replacement surgery (male patient, 53 years old), then cultivated after 2 and 4 passages, and frozen as stock samples at −80 °C in DMSO-containing cell freezing medium. Sample collection was always carried out with prior patient consent, which was reviewed and approved by the ethics committee (approval no. 1307/05).

For this work, the cells were thawed, cultured in osteogenic proliferation medium (OPM) and maintained under standard cell culture conditions. Cells were passaged when they reached about 80% confluence, then prepared as a single-cell suspension for adhesion assays where the cells are seeded at a concentration of 1 × 106 cells/ml. Detailed culture conditions are described in a previous work [32].

In the assay, a 30 µL drop of the cell suspension was added to specimen surfaces in a well plate and left in the incubator for 2 h to allow for initial adhesion before more OPM (300 µL) is added. The specimens are incubated for another 22 h, then washed with PBS to remove loosely adhered cells. The cells on the specimens were then fixed with 4% paraformaldehyde solution and analyzed under LSM.

### Surface characterization

Surface topography plays a crucial role in adhesion and proliferation of bacteria as well as eukaryotic cells [33–35]. Therefore, it is crucial to characterize the coating.

#### Surface roughness

The surface profiles were measured using LSM. From the profiles, surface roughness R_a_ and waviness W_z_ and W_sm_ were extracted according to the ISO 4287, where W_z_ refers to the amplitude and W_sm_ refers to the wavelength of the waviness profile. The surface roughness is a characteristic measure for the surface microstructure, while the waviness characterizes the macrostructure which is controlled by the manufacturing process. Due to the characteristic anisotropic structures formed by the tool path during FFF 3D printing, these properties were measured in two directions: parallel (0°) and perpendicular (90°) to the polymer strands. An exemplary measurement is described in Fig. S3, where a differentiation between surface roughness and waviness is shown.

#### Surface wettability

The wettability of the surfaces was assessed using water contact angles. Here, the images of a 5 µL water droplet placed on each specimen are taken with a Dino-Lite digital microscope (Dino-Lite Europe, Almere, the Netherlands) in accordance with DIN EN 828 and assessed with Drop Snake Analysis [36] on ImageJ.

#### Adhesion of the coating to PEEK substrate

The adhesion of the coatings to the PEEK substrate was evaluated using a manual cross-cut test, following the standard DIN EN ISO 2409 with a subsequent tape test. 2 sets of 6 parallel lines are made perpendicular to each other on the surface with a sharp cutter knife creating a grid pattern. A piece of standard transparent tape is then applied with light pressure onto the cross-cut area and subsequently removed in a fast motion. The test is repeated 4 times on different areas of the same specimen and visualized with the Dino-Lite digital microscope as well as LSM for higher resolution and further evaluation.

#### Cross-section image and chemical composition

The cross-sectional images of the coatings on 3D-printed PEEK substrate were taken and evaluated for their chemical compositions with ULTRA plusField Emission Scanning Electron Microscope (SEM) with an Everhart-Thornley secondary electron detector (Carl Zeiss, Oberkochen, Germany). Prior to the imaging, the printed and coated specimens were cut with WELL Diamond Wire Saws SA (Mannheim, Germany) Model 3500 to reveal the cross-section and polished with Ion Milling System ArBlade 5000 (acceleration voltage: 4 kV, discharge voltage: 1.5 kV, gas flow: 1 cm^3^min^−1^, table mode: Slow 30; Hitachi High-Tech Europe, Krefeld, Germany). The surface of the specimen was sputtered with approx. 70 nm platinum layer before ion milling in order to ensure a conductive contacting surface and to distinguish the original specimen from artifacts resulting from specimen preparation. Imaging was performed at an acceleration voltage of 0.8 kV in high vacuum at 10^−6^ mbar.

The composition of the specimen cross-section was analyzed by means of an Energy Dispersive X-Ray (EDX) (Oxford Instruments Ltd., Abingdon, UK) with Ultim® Max Silicon Drift Detector. The observed surface was coated with a 10 nm carbon layer in order to prevent charging of the probe. Here, the applied acceleration voltage was 10 kV.

#### Release rate of antimicrobial metal

The release of zinc ions (Zn^2+^) from the samples was measured over 5 days (120 h) in PBS at room temperature (20 °C) using inductively coupled plasma optical emission spectroscopy (ICP-OES) (Agilent 5800, Agilent Technologies Deutschland, Waldbronn, Germany). Sample collection was performed using a serial renewal method, as described in a previous work [25]. PEEK specimens with Zn coating were incubated with 10 mL 1x PBS in a well plate, and at defined timepoints (0, 0.5, 1, 2, 4, 8, 24, 48, and 120 h) the PBS medium is collected and the wells are replenished with 10 mL fresh PBS. The collected PBS was filtered through a 0.45 µm syringe filter (Minisart® NML, Sartorius, Göttingen, Germany) to remove larger particulate agglomerates, after which the filtrate was diluted to a final volume of 20 mL for ICP-OES analysis. Zn^2+^ concentration in each interval solution was quantified, and the cumulative ion release was calculated by summing the amounts released in all preceding intervals, normalized to the surface area of the sample.

### Statistical analysis

Quantitative results are expressed as mean ± standard deviation. Statistical significance between groups was evaluated using two-tailed *t* tests, with *p* values ≤ 0.05 being considered statistically significant. When not otherwise specified, asterisks (*) in tables and diagrams indicate statistical significance in comparison to uncoated PEEK control group (* for *p* ≤ 0.05, ** for *p* ≤ 0.01, *** for *p* ≤ 0.001).

## Results and discussion

### Biological investigations

#### Bacterial adhesion

**Fig. 4:**
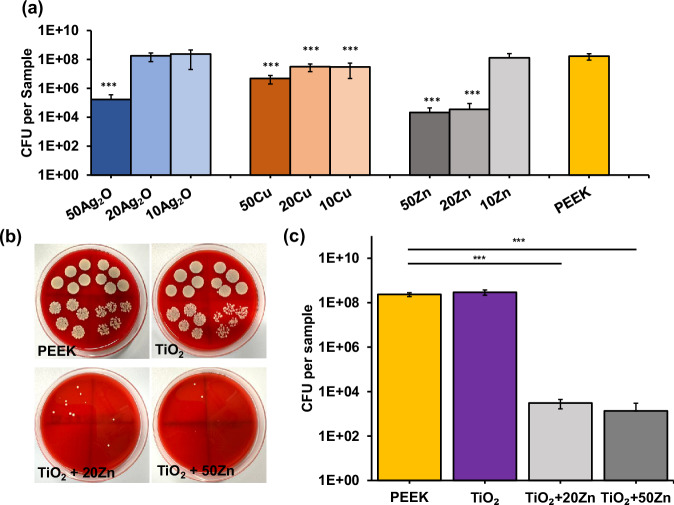
**a** Average bacteria count on PEEK specimens coated with 50 nm, 20 nm and 10 nm Ag_2_O, Cu and Zn coatings compared to uncoated PEEK (*n* = 3). **b** Digital photographs of representative agar plates with bacteria cultures taken from PEEK, TiO_2_, TiO_2_ + 20Zn and TiO_2_ + 50Zn specimens. Each agar plate contains 1st to 4th dilution step. **c** Average bacteria count from TiO_2_, TiO_2_ + 20Zn and TiO_2_ + 50Zn coatings compared to uncoated PEEK (*n* = 8)

In the first bacterial adhesion assay, the antimicrobial activity of Ag_2_O, Cu, and Zn coatings was evaluated at thicknesses of 10, 20, and 50 nm, with results summarized in Fig. 4a. In the following, the number before the element in the label refers to the corresponding coating thickness (in nm) of the element. At these thicknesses, Zn coatings demonstrated superior antimicrobial efficacy compared to Ag_2_O and Cu. Among all specimens, 50Zn achieved the highest bacterial reduction, with a CFU reduction of 3.9 log. 20Zn also exhibited strong antimicrobial performance with a reduction of 3.7 log, indicating that Zn maintains its effectiveness against *S. aureus* at lower thickness of 20 nm. However, 10Zn showed a decline in performance, suggesting that a minimum thickness of 20 nm is necessary for antimicrobial activity. In comparison, Ag_2_O and Cu coatings displayed less consistent antimicrobial activity. While 50Ag₂O reduced CFU by 3.1 log, thinner coatings (20Ag₂O and 10Ag₂O) were ineffective. Cu coatings exhibited moderate activity, with 50Cu achieving a 1.57 log reduction, and thinner coatings (20Cu and 10Cu) maintaining slight antimicrobial properties, achieving reductions of approximately 0.7 log.

In the second bacterial adhesion assay, the Zn coating is tested again in combination with 200 nm TiO_2_ as a base layer, with the results presented in Fig. 4c. Consistent with the earlier experiment, the 20 nm and 50 nm Zn layers exhibited strong bactericidal effects against *S. aureus*, achieving bacterial reductions of 4 log and 5 log, respectively. In contrast, the TiO_2_ layer alone did not display any bactericidal effects and even showed a slight, statistically insignificant increase in CFU. Representative images of the plated bacterial colonies from each sample group are shown in Fig. 4b.

**Fig. 5:**
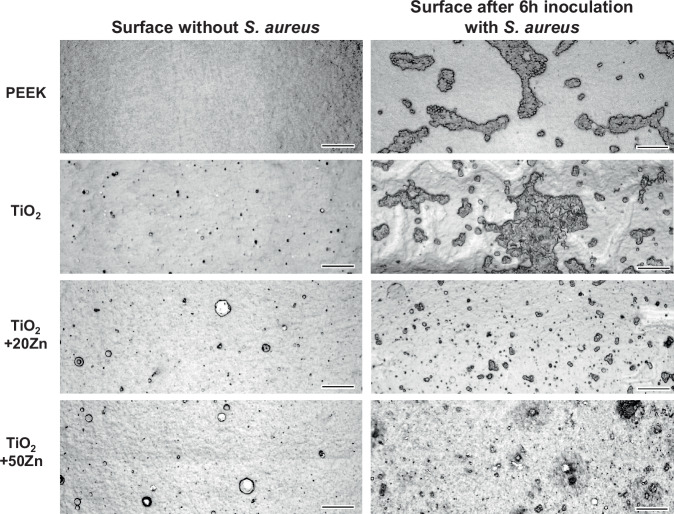
LSM images of the surfaces of uncoated PEEK and PEEK specimens coated with TiO_2_ + 20Zn and TiO_2_ + 50Zn. Left column shows the surfaces as fabricated. Right column shows the surface after inoculation with S. aureus for 6 h (scale bar corresponds to 10 µm)

The surface images taken after 6 h of bacterial inoculation are shown in Fig. 5. On both the PEEK substrate and the TiO_2_ coating, large bacterial aggregates can be observed scattered across the surface. These clusters represent the early stages of biofilm formation, in which bacteria adhere to each other and to the substrate while becoming embedded in a self-produced extracellular polymeric matrix that provides protection and structural stability. Although individual bacteria are difficult to distinguish due to surface roughness and the presence of macroparticles, the Zn-coated surfaces exhibit noticeably fewer and smaller biofilm regions. This suggests reduced bacterial adhesion and inhibited biofilm development compared with the other samples. The qualitative observations are consistent with the results of the quantitative bacterial adhesion assay.

#### In vitro cytotoxicity to fibroblastic cells

**Fig. 6:**
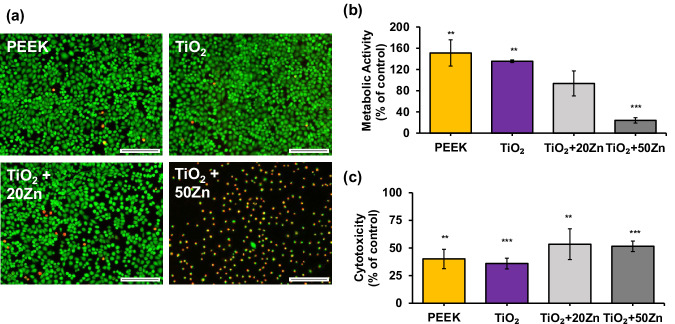
**a** Live/Dead™ staining of the fibroblast cells after contact with the specimens. Green stain (Calcein AM) indicates viable cells and red stain (EthD) indicates dead or damaged cells (scale bar corresponds to 10 µm). **b** Metabolic activity measured by WST-1 assay expressed in percentage to positive control (*n* = 5, * indicates statistically significant difference to positive control). **c** Cytotoxicity measured by LDH-Glo™ expressed in percentage to positive control (*n* = 5, * indicates statistically significant difference to positive control)

The biocompatibility of different coatings (uncoated PEEK, TiO_2_, TiO_2_ + 20Zn, and TiO_2_ + 50Zn) was evaluated through Live/Dead™ fluorescent staining, WST-1 assay, and LDH assay. Fluorescent images (Fig. 6a) reveal that PEEK and TiO_2_ materials exhibit predominantly green-stained cells, indicative of high cell viability. In contrast, TiO_2_ coated with Zn shows a thickness-dependent increase in red-stained cells, representing a reduction in viability. TiO_2_ + 20Zn shows a moderate presence of red-stained cells, while TiO_2_ + 50Zn shows a significant increase in red fluorescence, which implies a substantial cytotoxic effect with higher Zn coating thickness.

The WST-1 assay results (Fig. 6b) confirm these observations by quantifying the metabolic activity of the cells. PEEK and TiO2 maintain high metabolic activity, signifying excellent biocompatibility. However, the addition of Zn to TiO_2_ significantly reduces metabolic activity of the cells. The addition of 20 nm Zn leads to a slight decline in metabolic activity compared to pure TiO2 coating, while 50 nm Zn exhibits a dramatic decline, which implies strong cytotoxic effects of higher zinc content. Despite the adverse effects on the cells. TiO_2_ + 20Zn possesses over 80% viability of cells compared to control group, which, according to ISO 10993-5, is not considered cytotoxic.

The LDH assay (Fig. 6c) provides additional evidence of the cytotoxic effects of Zn layer. Both uncoated PEEK and TiO_2_ exhibit lower LDH release, hinting at lower cytotoxicity and good biocompatibility. TiO_2_/Zn combinations show higher LDH release at approximately 50% of positive control. However, the LDH release from PEEK and TiO_2_ samples raises concerns about the legitimacy of the results and accuracy of this assay

#### In vitro osteoblast adhesion

**Fig. 7:**
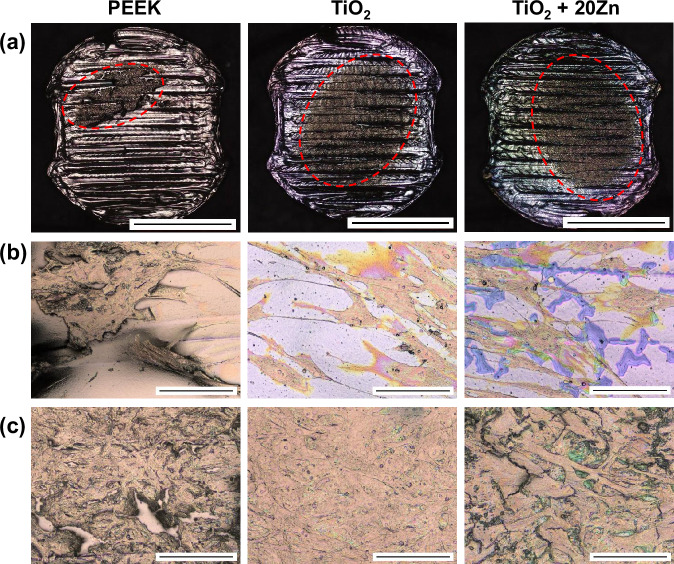
**a** Images of the specimens after osteoblast colonization and fixation. The matt brown areas (marked with red dotted circles) are the osteoblast film that adhered to the surface. On the uncoated PEEK, this area is significantly smaller and shows delamination, implying a suboptimal adhesion of the osteoblasts to the surface. (scale bar corresponds to 5 mm). **b** LSM images taken at the edge of the cell film, showing morphology of single cells (scale bar corresponds to 100 µm) **c** Images taken at the center of the cell film (scale bar corresponds to 100 µm)

Figure 7a shows images of the specimens after incubation with 30 µL drop of osteoblast cell suspension and subsequent fixation. Due to a technical issue, the TiO_2_ + 50Zn sample could not be fabricated in time and was therefore excluded from this test. The cell film on pure TiO_2_ and TiO_2_ + 20Zn coatings appears mostly intact, whereas the cell film on bare PEEK is detached, displaying noticeable gaps and voids.

The magnified images offer a more detailed examination of the cell films. To obtain a comprehensive understanding, images were captured from both the edges and the center of the cell films (Fig. 7b, c, respectively), allowing for an evaluation of the overall cell coverage and adhesion. On the PEEK specimens, the cell film has clearly delaminated, indicating that while the cells bond strongly to each other, they struggle to adhere effectively to the PEEK surface. In contrast, the TiO_2_ specimens show a dense and strongly adhered cell film. The TiO_2_ surface is entirely covered, with minimal tearing or voids observed in the cell film, suggesting strong adhesion and high cellular proliferation, which is a prerequisite for ingrowing tissue applications. On the TiO_2_/Zn coating, although no delamination is observed, the cell film is less densely packed compared to the pure TiO_2_ coating. This aligns with the results of the cytotoxicity test, which indicate that the presence of Zn in the coating layer can cause slight toxicity to cells, potentially reducing cell density and proliferation.

### Surface characterization

#### Surface roughness and wettability

**Fig. 8:**
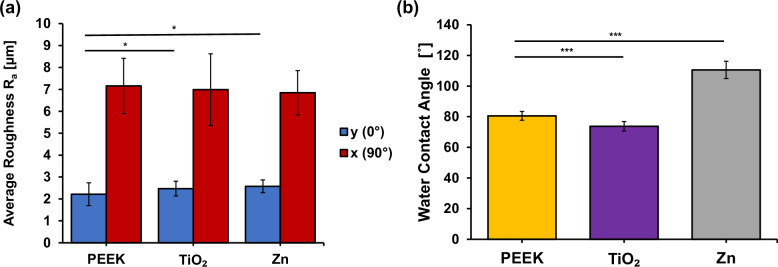
**a** Surface roughness (R_a_) of the specimens (*n* = 3) with different coating measured in 2 orientations; parallel (0°, blue bars) and orthogonal (90°, red bars) to the printed strands. **b** Contact angles of water droplet on uncoated PEEK specimens and specimens with different metallic coatings (*n* = 6). All specimens have a coating thickness of 50 nm

Figure 8a shows the surface roughness of 3D-printed PEEK structures with TiO_2_ and Zn coatings, measured in two orientations: 0° and 90°. The results show only small to no significant difference in average roughness (R_a_) between the type of coating applied. Overall, the roughness across all specimen groups averages to 2.3 ± 0.4 µm and 6.9 ± 1.3 µm in 0° and 90° direction, respectively. The difference in R_a_ values between the two measurement directions is due to the anisotropic nature of the 3D-printed surface.

The waviness in the 90° direction across all samples was measured to have an average amplitude (W_z,90°_) of 142.6 ± 32.5 µm and an average periodic wavelength (W_sm,90°_) of 481 ± 112 µm, which is in-line with the pre-configured line width of 450 µm. Meanwhile, the waviness in the 0° direction shows W_z,0°_ = 8.7 ± 2.8 µm and W_sm,0°_ = 214.6 ± 192.7 µm.

The resulted water contact angles are summarized in Fig. 8b. Despite the minimal variation in surface roughness, significant changes in wettability were observed. PEEK has a mean water contact angle of 80.5 ± 2.5˚. The lowest mean water contact angle can be observed in the TiO_2_ group at 73.8 ± 3.5˚. The Zn group presents the highest mean contact angle of 110.6 ± 5.6˚. Images of representative drop analysis are shown in Figure S4.

#### Adhesion of the coating to PEEK substrate

**Fig. 9:**
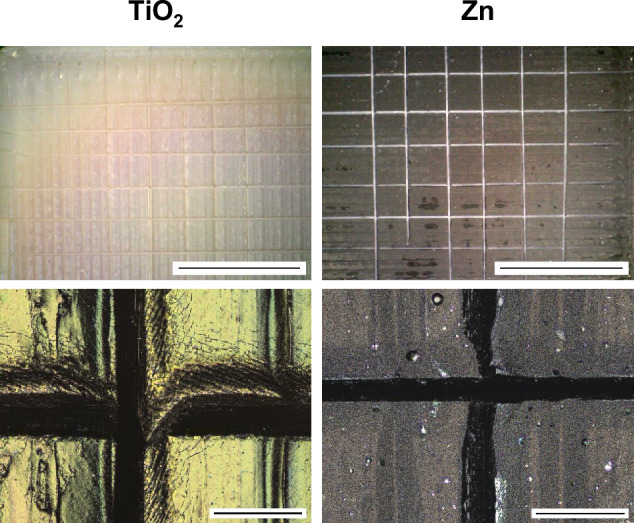
LSM images of PEEK specimens with TiO_2_ and Zn coatings after crosscut and tape-test (scale bar corresponds to 5 mm for top row and 200 µm for bottom row)

Figure 9 shows the images of the samples with TiO_2_ and Zn coating after a crosscut test followed by a tape test. The results suggest that all coatings exhibited strong adhesion with no delamination. There is no visible flaking or detachment observed in the coatings. In the case of the TiO_2_ specimen, microscopic fractures were observed around the crosscuts at high magnification, but no flaking occurred. As a result, the coatings were assigned to the highest crosscut grading (Gt0), indicating excellent adhesion to the PEEK substrates.

#### Cross-section image and chemical composition

**Fig. 10:**
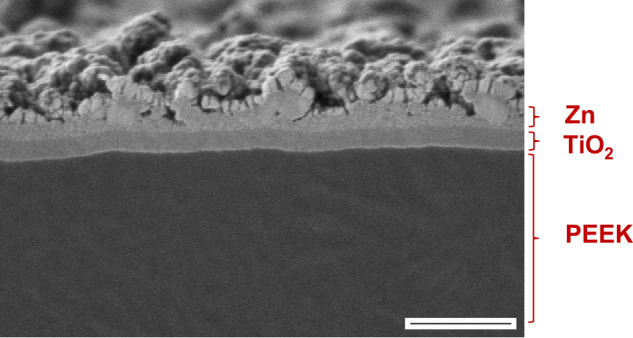
Cross-section image of the TiO2/Zn coating combination showing clear transitions between the layers. The uppermost layer (pale layer above Zn) is a layer of platinum, which was sputtered on to the specimen in order to ensure conductivity and to easily identify the top of the specimen during the EDX analysis (image acquired at 0.8 kV in high vacuum; scale bar corresponds to 500 nm)

SEM image of the cross-section of an exemplary sample is provided in Fig. 10. Here, a combination of TiO_2_/Zn coating on PEEK is depicted. The image shows the architecture of the material, with each layer serving a specific function: PEEK provides fundamental structure, TiO_2_ enhances bioactivity, Zn introduces additional antimicrobial effect. It can be observed that the two coating layers exhibit distinct structural characteristics. For TiO_2_, the layer appears to be even and homogenous throughout the surface. On the other hand, Zn layer has clumps and pores, which are likely attributed to its higher water contact angle observed in the previous section. This is further confirmed by the LSM images in the left column of Fig. 5, where a higher number of macroparticles in Zn layer can be observed. The higher number of macroparticles is likely attributed to the lower melting point of Zn compared to Ti, since the material can be melted and ejected from the cathode spot more easily under the heat of the vacuum arc.

**Fig. 11:**
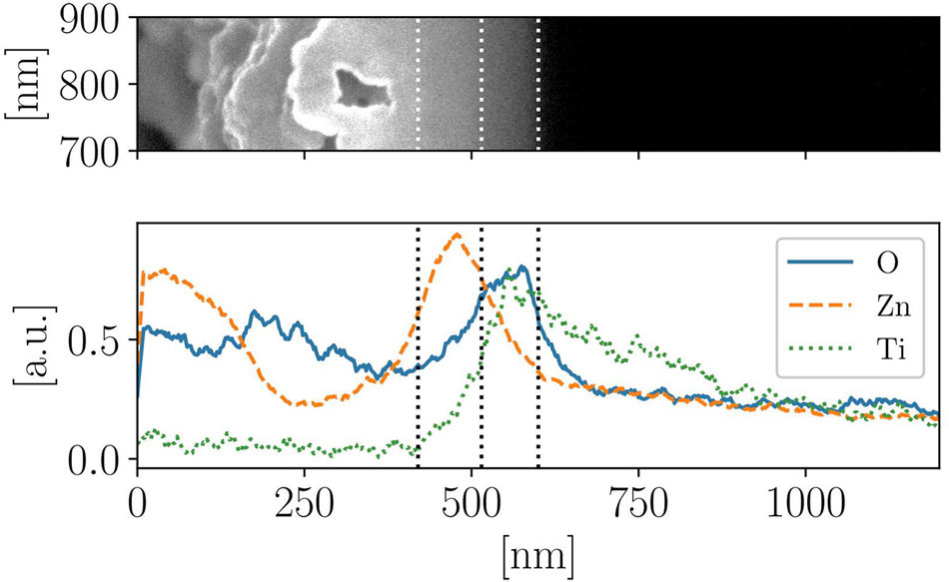
Evaluation of element distribution detected using EDX: SEM micrograph of the region of signal accumulation (top) and vertically accumulated and normalized signals of detected characteristic element lines for O (solid, blue), Zn (dashed, orange) and Ti (dotted, green) (bottom)

Figure 11 evaluates the coating composition, measured by EDX. Here, the measurement was performed with an acceleration voltage of 10 kV ensuring sufficient excitation of Ti atoms in the coating matrix. However, this leads to a decreased lateral resolution up to 1 µm, depending on material combination [37], therefore only a qualitative evaluation is possible. Here, the True Map signal reconstruction provided by AZtecOne Software (Oxford Instruments, UK) is used in order to identify the location of the elements. Lineout signals of different characteristic lines show qualitative distribution of Zn, Ti and O depending on the vertical position in the cross-section. The signal from 0 to ca. 400 nm is attributed to the specimen surface. The region marked with white and black dashed lines between ca. 400 and 500 nm corresponds to the previously optically identified layer in the SEM micrograph, where Zn is present. The peak concentrations of O and Ti are located in the subsequent layer (between ca 500 and 600 nm), which was previously identified as TiO_2_. Quantitative estimation of atomic fraction in the region of highest Ti and O concentrations confirms the TiO_2_ stoichiometry, while Zn is assumed to be mostly unoxidized.

#### Release rate of Zn coating

**Fig. 12:**
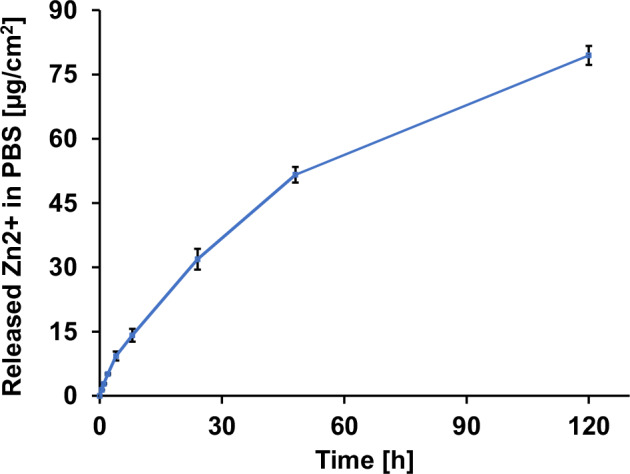
Cumulative release of Zn²⁺ ions from the coating into PBS over 120 h (*n* = 4)

The cumulated release of zinc ions from the 500 nm Zn coating into PBS increased steadily throughout the 120 h observation period (Fig. 12). The high coating thickness was selected to enable monitoring of zinc dissolution over an extended duration while maintaining concentrations within the detectable range. A rapid initial release was recorded within the first 24 h, followed by a slower but continuous increase, reaching a total release of approximately 80 µg/cm² after 120 h. During the first 4 h, the Zn²⁺ ion concentration in the solution increased linearly, indicating a constant release rate during this initial phase.

## Discussion

In the previous section, the coatings were evaluated for their physical characteristics using various methods. Surface measurements revealed minimal to no significant topographical differences between the coated specimens and the uncoated PEEK reference, which can be attributed to the coatings being extremely thin and homogeneous. The vacuum arc deposition process generates a plasma plume that uniformly deposits metal-based coatings onto the substrate, maintaining the original surface roughness. Minor variations in roughness values were likely caused by artifacts generated by the printing process itself or macroparticles generated by the coating source, which could potentially be eliminated through further optimization of the system (magnetic filtering) [38]. The waviness of the surface, on the other hand, could be adjusted to a desired value by choosing the right printing parameters. Roughened as well as porous surfaces have been shown in literature to promote the adhesion of cells [39–41]. Within the bounds of common slicer softwares, the waviness in the 90° direction can be easily manipulated by the choice of layer height, nozzle diameter, strand width and strand distance. The waviness in the 0° direction is influenced mainly by characteristics of the extruder stepper motor and the printing speed. Further modification of the surface topography can be explored with advanced slicing method such as FullControl [42].

There is currently no established consensus in the literature regarding the optimal surface roughness for the bone-implant interface. Surface roughness between 3–5 µm is preferred over smoother surfaces (R_a_ < 1 µm), since this range of roughness has been shown to promote clot adhesion, protein binding, and key growth factors for osteoblasts [43–45]. The surface roughness measured in this study lie within a similar range of around 2–7 µm, which should sufficiently support the adhesion and proliferation of osteoblasts. However, higher surface roughness values (R_a_ > 10 µm) have also been shown in literature to improve osteoblast activity [46–48]. Others have found only slight or no change in cell behavior at different surface roughnesses [49]. Due to these conflicting data in the literature, surface roughness should be considered in conjunction with other factors, such as the surface’s chemical composition and the morphology of its topographic ridges and grooves [35]. Studies have stated that the ridges and grooves on the surface should provide enough space to facilitate the cells and their subsequent spread [50, 51]. Grooves smaller than the cell size lead to lower cell adhesions. Considering the cell size of human osteoblasts ( ~ 50 µm in diameter before adhesion), the grooves or cavities on the surface are recommended to be wider than 50 µm in order to sufficiently support adhesion [52]. In this study, the surface grooves measured approximately 240 µm wide (half of the waviness W_sm_,90°) and 71 µm deep (half of the waviness W_z_,90°). Moreover, there is a general consensus for porous structure to possess a pore size between 400–600 µm in order to optimize the ingrowth of osteoblast cells and vascularization [41, 53, 54]. This range is easily achievable with an FFF 3D printer. While porosity is an important factor for specific biomedical applications, this study focused on demonstrating the technology and the potential of surface coatings. Therefore, the porosity of the printed parts was not further examined.

The contact angle measurements for PEEK in this study were consistent with values reported in the literature [55, 56]. Despite the minimal variation in surface roughness, significant changes in wettability were observed. The TiO_2_ coating increased wettability, whereas the Zn coating significantly decreased it. Taken together with surface roughness measurements, the results suggest that the changes in wettability here are predominantly influenced by surface chemistry rather than surface roughness. The increased wettability through TiO_2_ coating is particularly promising for addressing the bioinert nature of PEEK surfaces. A more hydrophilic surface has been shown to improve cell interaction, proliferation, and adhesion, thereby potentially accelerating bone healing, enhancing osteogenic capacity, and improving implant osseointegration [56, 57].

In the case of microorganisms, the relationship between roughness and bacterial adhesion is usually explored in sub-micron range and also has conflicting findings [58]. Nevertheless, the results from our biological assessments indicate that the thin Zn film produced by pulsed vacuum arc deposition exhibits excellent antimicrobial properties. However, the thickness of the film significantly influenced its bactericidal effectiveness. We have identified the antimicrobial efficacy threshold of pure Zn layer against *S. aureus* to be approximately of 20 nm thickness. The use of TiO_2_ as a base layer enhances the bioactivity of PEEK, as TiO_2_ has proven to be a superior substrate for osteoblast to adhere and proliferate on. By combining TiO_2_ and an antimicrobial agent like Zn in layered structures, the risk of early infections following implantation can be effectively minimized. In this configuration, the top Zn layer would release ions over time, gradually diminishing and exposing the TiO_2_ layer underneath, which then facilitates osteoblast integration during the recovery phase.

The dissolution behavior confirms a release-based antibacterial mechanism of the Zn coating. The initial phase shows a rapid release of Zn^2+^, followed by a slower but steady increase at later time points. The reduced release rate in the later stages may partly result from local saturation effects that develop between medium exchanges, as the intervals between measurements were longer. The antimicrobial effect of metals, such as Zn, is typically attributed to the release of metal ions, which in turn generate reactive oxygen species (ROS) in the surroundings [59, 60]. These ROS attack bacterial membranes and damage the DNA, ultimately killing the bacteria. The use of metal-polymeric implants offers distinct advantages over drug-eluting alternatives, particularly in the context of rising antimicrobial resistance (AMR). Unlike antibiotic-releasing coatings, metal-based antimicrobial surfaces, such as those incorporating Zn, do not rely on specific biochemical pathways and are less likely to induce resistance development, making them broad-spectrum and durable over time [61].

While the release-based killing mechanism is highly effective against bacteria, the result has shown that it can also have adverse effects on eukaryotic cells. The cytotoxicity of dissolved Zn ions to the human cells has been observed in several studies [62–65]. Therefore, developing a balanced antimicrobial material requires a trade-off between efficient bacterial eradication and maintaining healthy proliferation of host cells, which must be carefully considered for biomedical applications. The mild cytotoxicity observed in the Zn coating is likely due to its strong initial ion release rate, which induces oxidative stress in cells [64]. The results demonstrate that a 20 nm Zn layer can deliver a short-term burst of zinc ions capable of exerting bactericidal effects while maintaining enough cytocompatibility to accommodate osteoblast adhesion, supporting its potential use as a functional implant coating to reduce infection risk during the early healing phase. The biological evaluation in this study was limited to a 24 h observation period. Long-term assessments of the coating’s effectiveness are currently being planned. While qualitative imaging confirmed improved osteoblast adhesion on TiO_2_ and TiO_2_/Zn-coated surfaces, the current study lacks quantitative data on adhesion strength. This metric is essential for comparing performance of the coatings and validating long-term biointegration potential. Therefore, future work will include quantitative assays to provide a more robust evaluation of osteoblast adhesion and proliferation.

Overall, the combination of TiO_2_ and Zn coatings demonstrated enhanced bioactivity, offering a promising solution that integrates both antimicrobial and osteointegrative properties. These findings are particularly important for addressing the bioinertness of PEEK, a known limitation for osseointegration, especially in load-bearing implants where long-term stability is essential. Moreover, this study opens the door to exploring various other coating combinations. In antibacterial tests, coatings of Ag_2_O and Cu have also shown potential in combating bacterial growth. Additionally, the use of high-temperature FFF technology provides flexibility in material processing. Beyond PEEK, a variety of other thermoplastic polymers can be processed using this manufacturing method with minimal modifications. One other significant advantage of AM is its ability to fabricate porous structures, which can enhance tissue integration. Investigating the coating of such porous architectures represents a valuable direction for future research in biomedical applications.

One of the key attributes of this work is the integration of the vacuum arc coating unit into the AM workflow, which simplifies the fabrication and coating process. We have successfully demonstrated the potential application of this manufacturing method in the field of biomedical engineering. By combining printing and coating in a single step within a vacuum environment, this hybrid approach can significantly reduce manufacturing time and costs while enhancing the overall functionality of medical implants. This streamlined process holds promise for scalable production, potentially allowing point-of-care manufacturing in clinical settings, reducing the dependency on centralized manufacturing facilities, and enabling personalized implants tailored for individual patient needs.

## Conclusions

This study demonstrates a novel hybrid additive manufacturing approach that integrates FFF 3D-printing with in situ vacuum arc plasma coating to enhance the performance of PEEK implants. We successfully deposited TiO_2_/Zn thin film coatings onto 3D-printed PEEK surfaces and showed that this method provides:

1. Robust antimicrobial activity: Zn coating significantly reduced bacterial adhesion (up to 5-log reduction against *S. aureus*).
2. Cytocompatibility: TiO_2_/Zn coatings maintained acceptable biocompatibility with fibroblasts and supported osteoblast adhesion, especially at lower Zn concentration.
3. Potential clinical benefits: This one-step process offers a scalable path for producing implants with antibacterial properties, making it highly relevant for orthopedic applications.

Overall, this integrated printing-coating strategy simplifies manufacturing while addressing two critical challenges in implantology: infection prevention and tissue integration. Future work will focus on optimizing release profiles, long-term performance, and extending this approach to other implantable polymers and porous architectures.

## Supplementary information

Supplementary Material

## Data Availability

Data will be made available on request.
