# Supplementary material for: Biofunctionalization of 3D printed PEEK using integrated cathodic arc plasma coating: a one-step solution to antimicrobial and bioactive PEEK Implant
Source: J Mater Sci Mater Med. 2025 Nov 21;36(1):109. doi: 10.1007/s10856-025-06971-7 (PMC12638338; doi:10.1007/s10856-025-06971-7)
Supplement: Supplementary file 1 — Supplementary Material [file 10856_2025_6971_MOESM1_ESM.docx]

Supplementary Material

3D-printed PEEK with Antimicrobial TiO2/Zn Coating Using Vacuum Arc Plasma Deposition

Jay Phruekthayanon ^1,^*, Marina Kühn-Kauffeldt ^1^, Marvin Kühn ^1^, Jörg Gregor Diez^3,4^, Stephan Heller ^2^, Jutta Tübel ^2^, Rainer Burgkart ^2^ and Andreas Obermeier ^2^

^1^ Institute for Electrical Energy Systems, University of Bundeswehr Munich, 85579 Neubiberg, Germany

^2^ Clinic for orthopedics and Sports Orthopedics, Klinikum rechts der Isar, TUM School of Medicine and Health, Technical University of Munich, 81675 Munich, Germany

^3^ Department 310-Surface Technology and Analytics, Wehrwissenschaftliches Institut für Werk- und Betriebsstoffe (WIWeB), 85435 Erding, Germany

^4^ Institute of Lightweight Engineering, University of the Bundeswehr Munich, 85579 Neubiberg, Germany


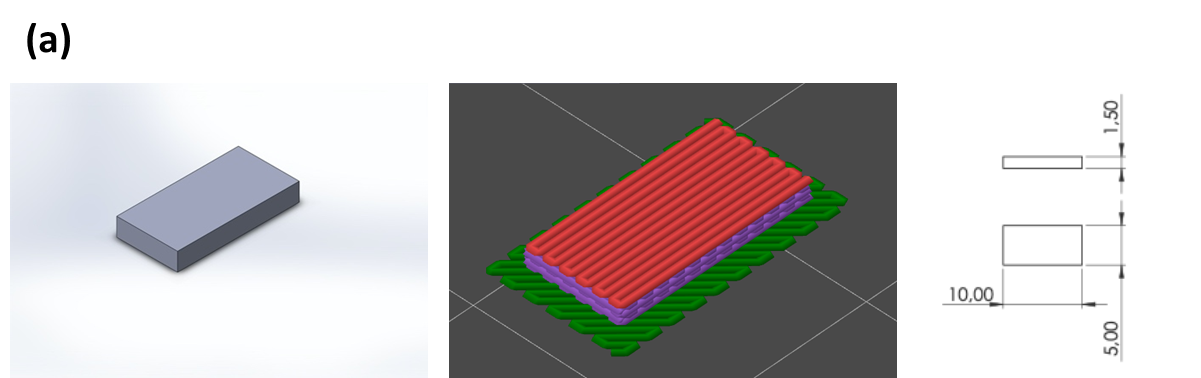


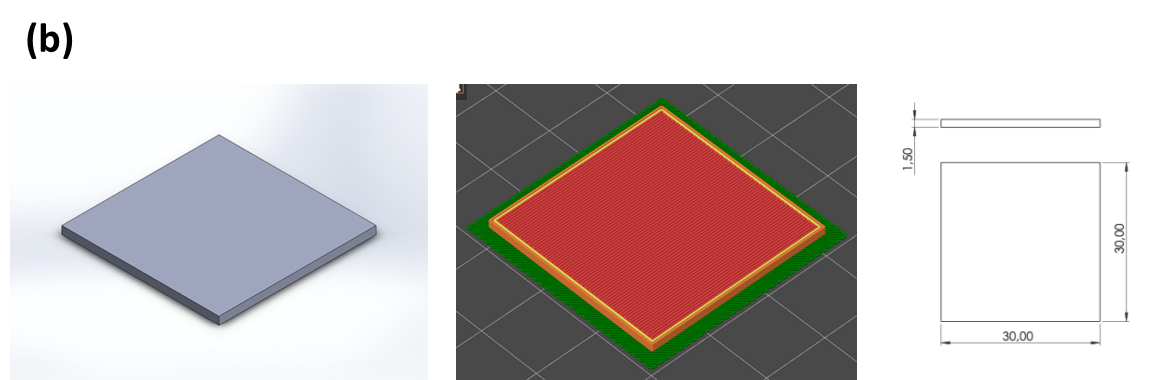


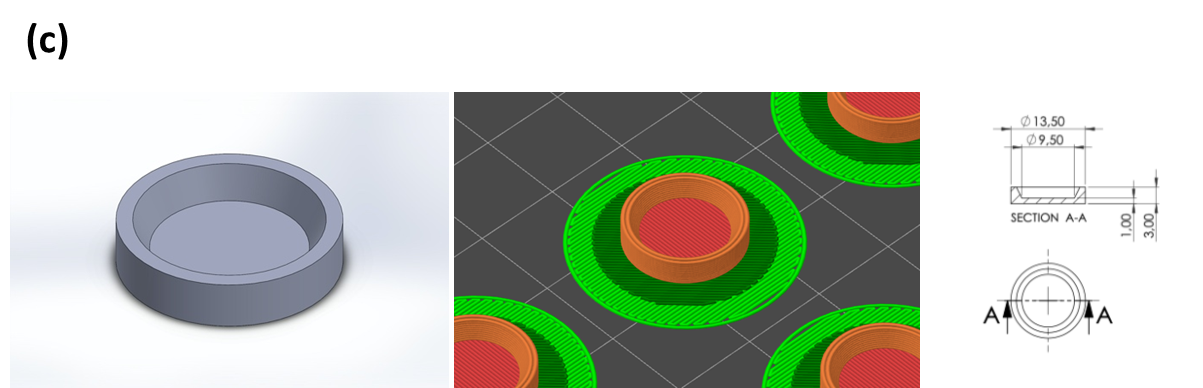


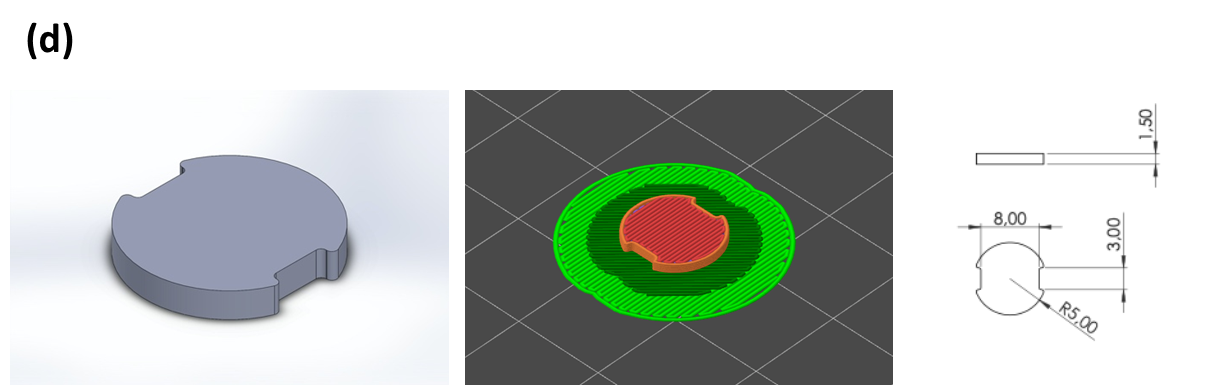


**Figure S 1.** CAD model and dimensions of each specimen type (a) specimen for roughness and water contact angle measurement (b) specimen for crosscut test (c) specimen for bacterial adhesion assay (d) specimen for cytotoxicity and osteoblast adhesion assays


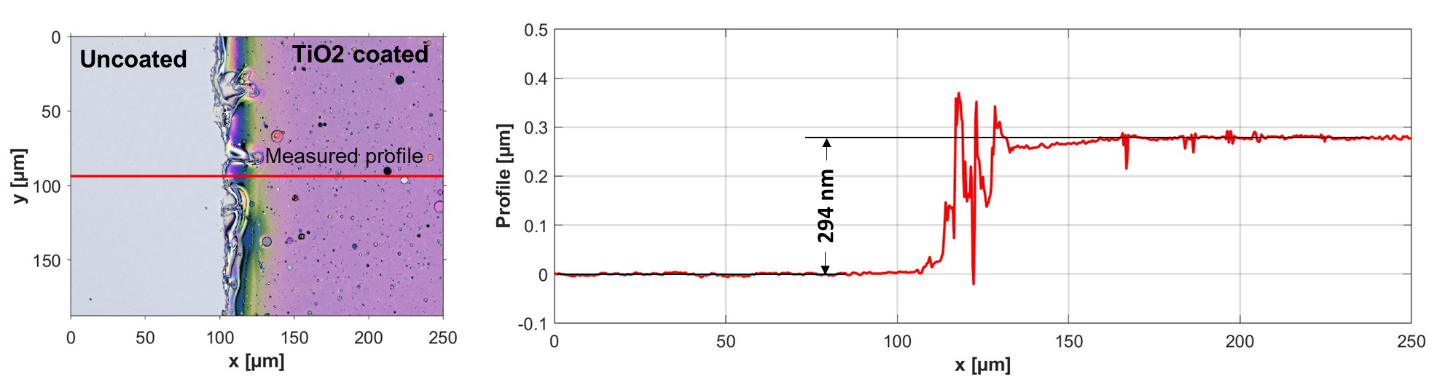


**Figure S 2.** Exemplary measurement of TiO_2_ coating thickness. The coating is deposited on a half-masked silicon wafer. After the mask was removed, a step profile can be observed with LSM.

**Table S 1.** Deposition rate of each coating at cathode-target distance of 20mm

| **Coating** | **Average deposition rate [nm/pulse]** |
| --- | --- |
| TiO_2_ | 0.25 |
| Ag | 0.19 |
| Zn | 0.53 |
| Cu | 0.12 |


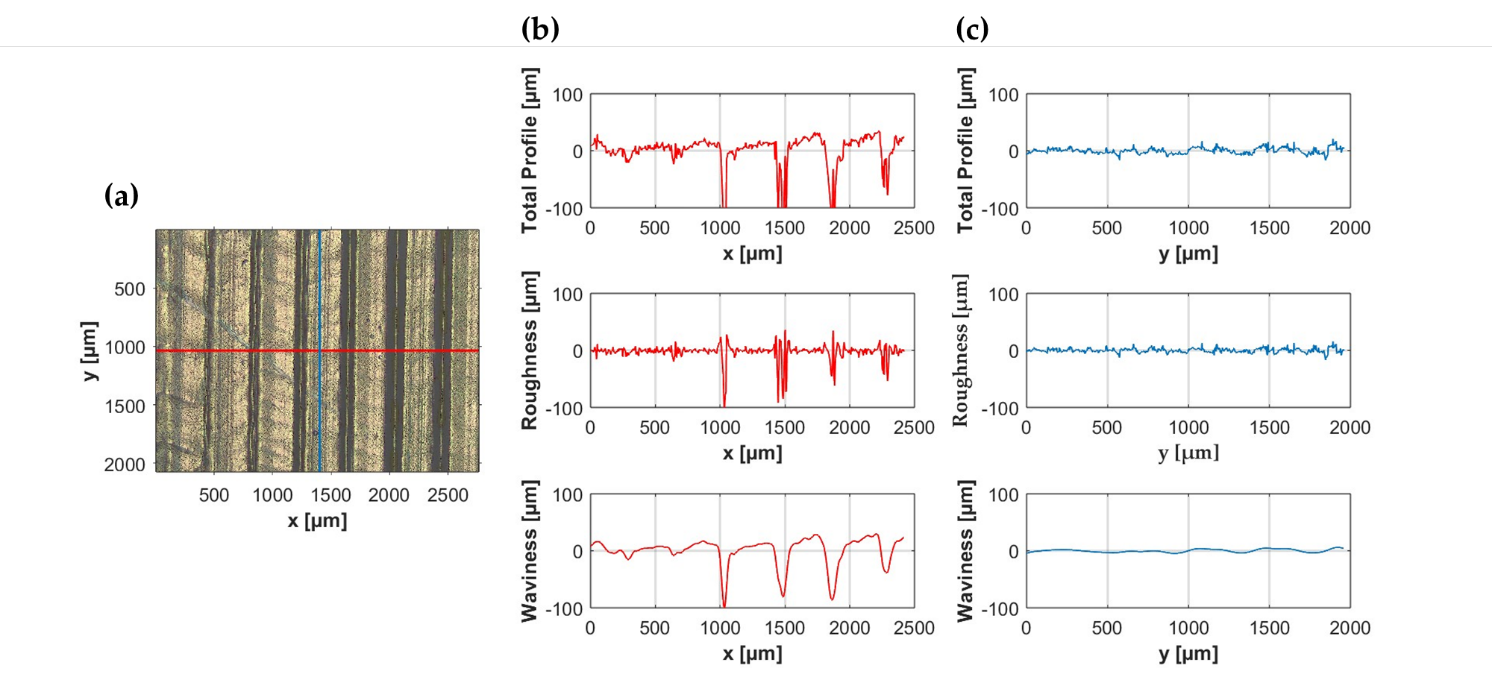


**Figure S 3.** Exemplary measurement of surface roughness and waviness in x- and y-direction. To isolate surface roughness from surface waviness, filters of λc,0°=0.25 mm for parallel and λc,90°=0.08 mm vertical directions were applied for measurements. A finer filter was used for the measurement in 90° direction due to the ridges between the strands. (a) LSM image of the surface (b) measurements of the surface profile in y-direction (90° to the strands) (c) measurements of the surface profile in x-direction (0° to the strands).


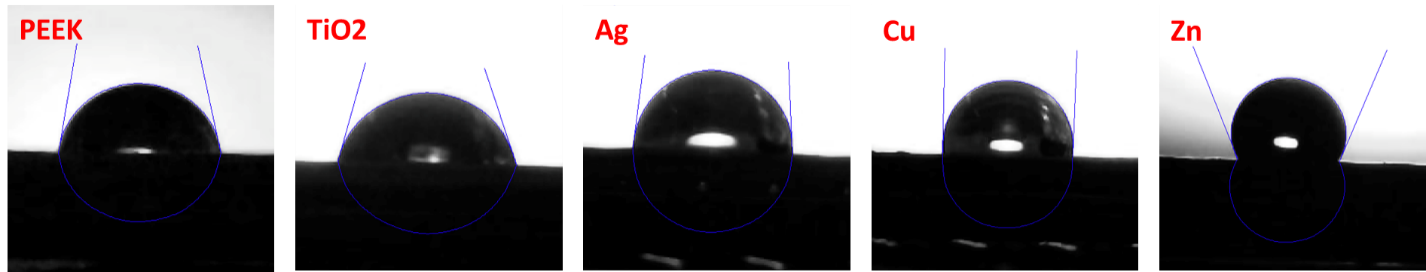


**Figure S 4.** Representative images of drop analysis for contact angle measurement
